# Identification and integration analysis of a novel prognostic signature associated with cuproptosis-related ferroptosis genes and relevant lncRNA regulatory axis in lung adenocarcinoma

**DOI:** 10.18632/aging.204561

**Published:** 2023-03-03

**Authors:** Tianyue Wang, Xinyu Jiang, Ying Lu, Yanmin Ruan, Jiamin Wang

**Affiliations:** 1 The Second Clinical Medical College, Zhejiang Chinese Medical University, Hangzhou 310053, China; 2 The First Clinical Medical College, Zhejiang Chinese Medical University, Hangzhou 310053, China; 3 The Fourth Clinical Medical College, Zhejiang Chinese Medical University, Hangzhou 310053, China

**Keywords:** ferroptosis, cuproptosis, prognostic signature, lncRNA, lung adenocarcinoma

## Abstract

Lung adenocarcinoma (LUAD) is a highly prevalent malignancy worldwide, and its clinical prognosis assessment and treatment is a major research direction. Both ferroptosis and cuproptosis are novel forms of cell death and are considered to be important factors involved in cancer progression. To further understand the correlation between the cuproptosis-related ferroptosis genes (CRFGs) and the prognosis of LUAD, we explore the molecular mechanisms related to the development of the disease. We constructed a prognostic signature containing 13 CRFGs, which, after grouping based on risk score, revealed that the LUAD high-risk group exhibited poor prognosis. Nomogram confirmed that it could be an independent risk factor for LUAD, and ROC curves and DCA validated the validity of the model. Further analysis showed that the three prognostic biomarkers (LIFR, CAV1, TFAP2A) were significantly correlated with immunization. Meanwhile, we found that a LINC00324/miR-200c-3p/TFAP2A regulatory axis could be involved in the progression of LUAD. In conclusion, our report reveals that CRFGs are well correlated with LUAD and provide new ideas for the construction of clinical prognostic tools, immunotherapy, and targeted therapy for LUAD.

## INTRODUCTION

Until 2022, lung cancer has the highest global mortality rate among cancers, killing about 350 people each day [1]. Non-small cell lung carcinoma (NSCLC) accounts for approximately 85% of all lung cancer patients [2]. The incidence of LUAD, the most predominant subtype of NSCLC, has been increasing year by year [3]. Although the clinical management strategies for LUAD are continuously updated, there are still problems such as low early diagnosis rates and unsatisfactory long-term survival of patients [4]. In this regard, there is an urgent need to find a new clinical model that can accurately diagnose and assess the prognosis of LUAD, and further explore the molecular mechanisms related to the development of the disease, which may provide a new idea for the subsequent targeted therapy.

Ferroptosis, a novel type of programmed cell death [5], distinguishes itself from other cell death modalities such as apoptosis and necrosis and occurs mainly due to the presence of divalent iron ions that accelerate the process of lipid peroxidation of saturated fatty acids in the body, resulting in oxidative stress in cells, which further induces cell death [6]. The iron metabolism has a dual effect on tumor development, i.e., an increase in iron content within a certain range is not conducive to the control of tumor cell growth and multiplication [7], but when the intracellular iron concentration exceeds a threshold, the ferroptosis effect that is triggered at this time has a positive effect on tumor control [8]. A number of studies have shown that various tumor suppressors can upregulate the sensitivity of tumor cells to iron death, such as p53 and BRCA1-Associated Protein 1 (BAP1) [9, 10], both of which can inhibit the expression of SLC7A11 coupled with ferroptosis mechanism to exert tumor suppressive effects. As an essential co-factor in the body, copper, similar to iron, plays an important role in biological processes such as participation in mitochondrial respiration and regulation of signaling pathways [11]. Cuproptosis death is a novel mode of cell death proposed by recent studies, and its mechanism of occurrence depends on intracellular copper accumulation [12]. During the development of cuproptosis, excess copper ion carriers were found to bind to fatty tricarboxylic acid cycle proteins to trigger protein aggregation, leading to acute proteotoxic stress [13]. The discovery of cuproptosis may become a new mechanism for the treatment of tumors in clinical practice, which will guide the future research direction of tumor diagnosis and treatment [14].

Current studies have confirmed the involvement of ferroptosis and cuproptosis in the development of many cancers and both are considered to be important factors strongly associated with cancer progression [15, 16]. It was found in clinical studies on LUAD that the occurrence of ferroptosis has significant implications for the treatment of patients with the advanced and drug-resistant diseases [17], in which cuproptosis also showed a positive prognostic effect [18]. Collectively, it is reasonable to speculate that the combination of the two death modalities may provide better control of tumor progression. Recent studies have shown that CRFGs have superior performance in prognosis and immune infiltration in hepatocellular carcinoma [19]. In the study about colorectal cancer, we can find that the prognostic significance of CRFGs in cancer has higher reliability [20]. The research results mentioned above prove that CRFGs are relevant in the exploration of clinical treatment of malignant tumors. However, the relevant studies of CRFGs in LUAD have not been addressed, thus the combined therapeutic mechanism of ferroptosis and cuproptosis in LUAD needs to be further discovered. Therefore, the study of CRFGs in LUAD may also provide new ideas for the diagnosis and treatment of this disease.

The lncRNA also plays a crucial role in the development of cancer by interacting with DNA, RNA, and proteins to regulate gene expression in both cis or trans transcription, the organization of nuclear structural domains and at the post-transcriptional level [21]. It is now generally accepted that lncRNA can identify cancer cell pathology and identify tumor subtypes, with important prognostic value, and is a biomarker for a variety of cancers, which can be used to guide patients in therapy selection [22]. In the available studies, lncRNA was found to have a significant impact on the progression and metastasis of LUAD. It has been demonstrated that the JPX/miR-33a-5p/Twist1 axis can activate Wnt/β-catenin signaling to promote metastasis in LUAD [23]. Meanwhile, LINC00472 was identified as a protective factor for LUAD [24]. In contrast, the molecular regulatory mechanism of CRFGs is still unclear, which enlightens us to conduct an in-depth investigation of lncRNA to lay the foundation for clinical studies of LUAD.

**Figure 1:**
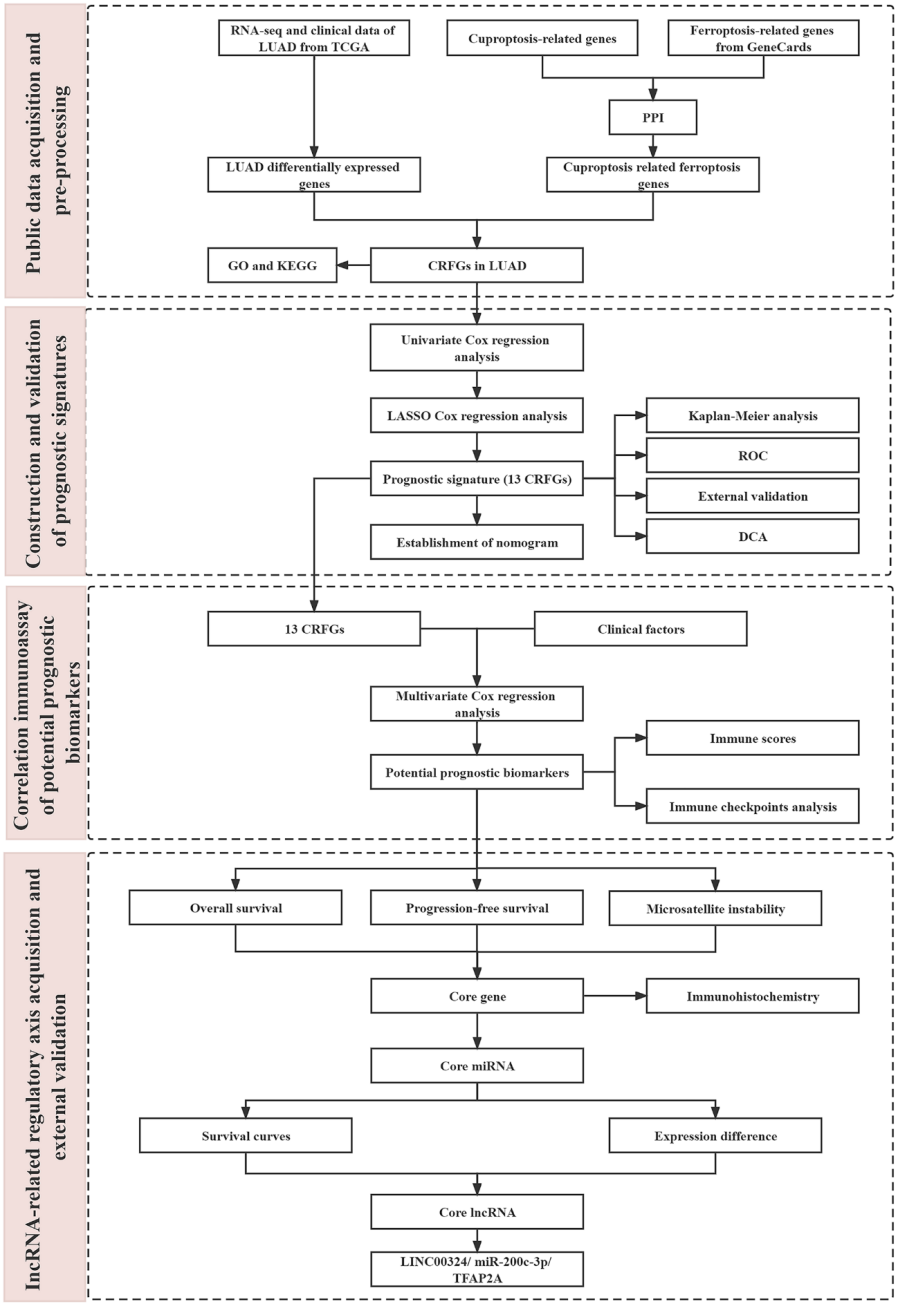
The flow chart of this study.

In the present study, we constructed a clinical prediction model about CRFGs in LUAD by bioinformatics using the gene signature of cuproptosis-related ferroptosis as the main object of analysis and validated it using an external dataset for correlation. Based on the model, we established potential prognostic biomarkers. We also further explored the regulatory mechanisms of the two combined death modes by related lncRNA and the potential impact on the prognosis of LUAD. The workflow of this study is shown in Figure 1.

## RESULTS

### Acquisition and enrichment analysis of CRFGs

In the Genecards database, 302 genes related to ferroptosis were obtained (Supplementary Table 1), and 16 genes (FDX1, LIPT1, LIAS, DLD, MTF1, GLS, CDKN2A, DLAT, PDHA1, PDHB, DBT, GCSH, DLST, SLC31A1, ATP7A, and ATP7B) closely related to copper death were collated from the latest studies on cuproptosis [13].

**Figure 2:**
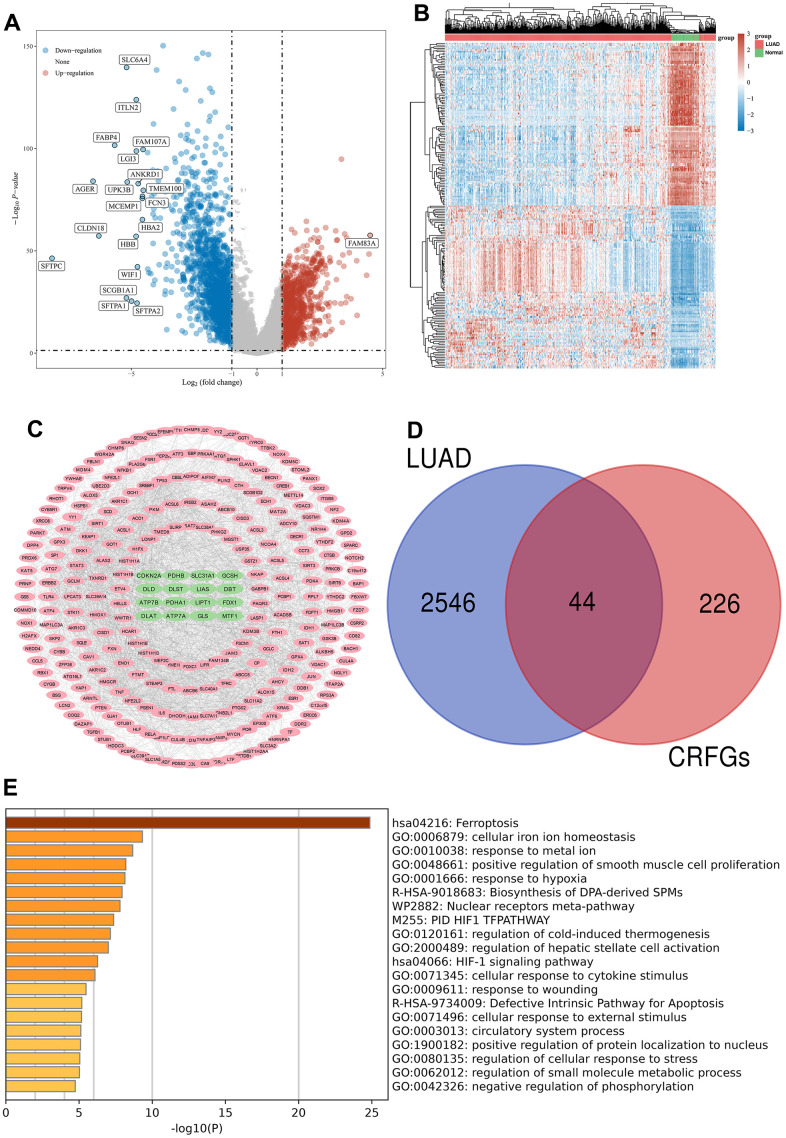
**Acquisition and enrichment analysis of LUAD-related CRFGs.** (**A**) Volcano plot, blue dots indicate genes significantly down-regulated in expression in LUAD samples compared to normal samples, and red dots indicate genes significantly up-regulated. (**B**) Heat map. (**C**) PPI network diagram of CRFGs, ferroptosis-related genes are shown with pink nodes and green nodes representing cuproptosis-related genes. (**D**) Venn diagram constructed by LUAD and CRFGs. (**E**) GO and KEGG enrichment analysis of LUAD-related CRFGs, with the longer columns representing higher enrichment.

Gene differential expression analysis was performed on 516 LUAD samples and 59 normal samples in the TCGA database, volcano maps (Figure 2A) and gene heat maps (Figure 2B) were constructed, and a total of 2590 genes showing differential expression were acquired, of which 928 genes were up-regulated in LUAD compared to normal samples, and the remaining 1662 genes were down-regulated (Supplementary Table 2).

The PPI network was constructed from the genes associated with ferroptosis and cuproptosis (Figure 2C), and the network contained a total of 270 nodes with 16 genes associated with cuproptosis and 254 genes associated with ferroptosis. Venn diagrams were plotted for the 2590 differential genes and 270 CRFGs in LUAD, and a total of 44 common genes were found (Figure 2D). The top twenty results with the highest gene enrichment in CRFGs in LUAD are shown in Figure 2E.

### Potential prognostic genes and mutational landscape

**Figure 3:**
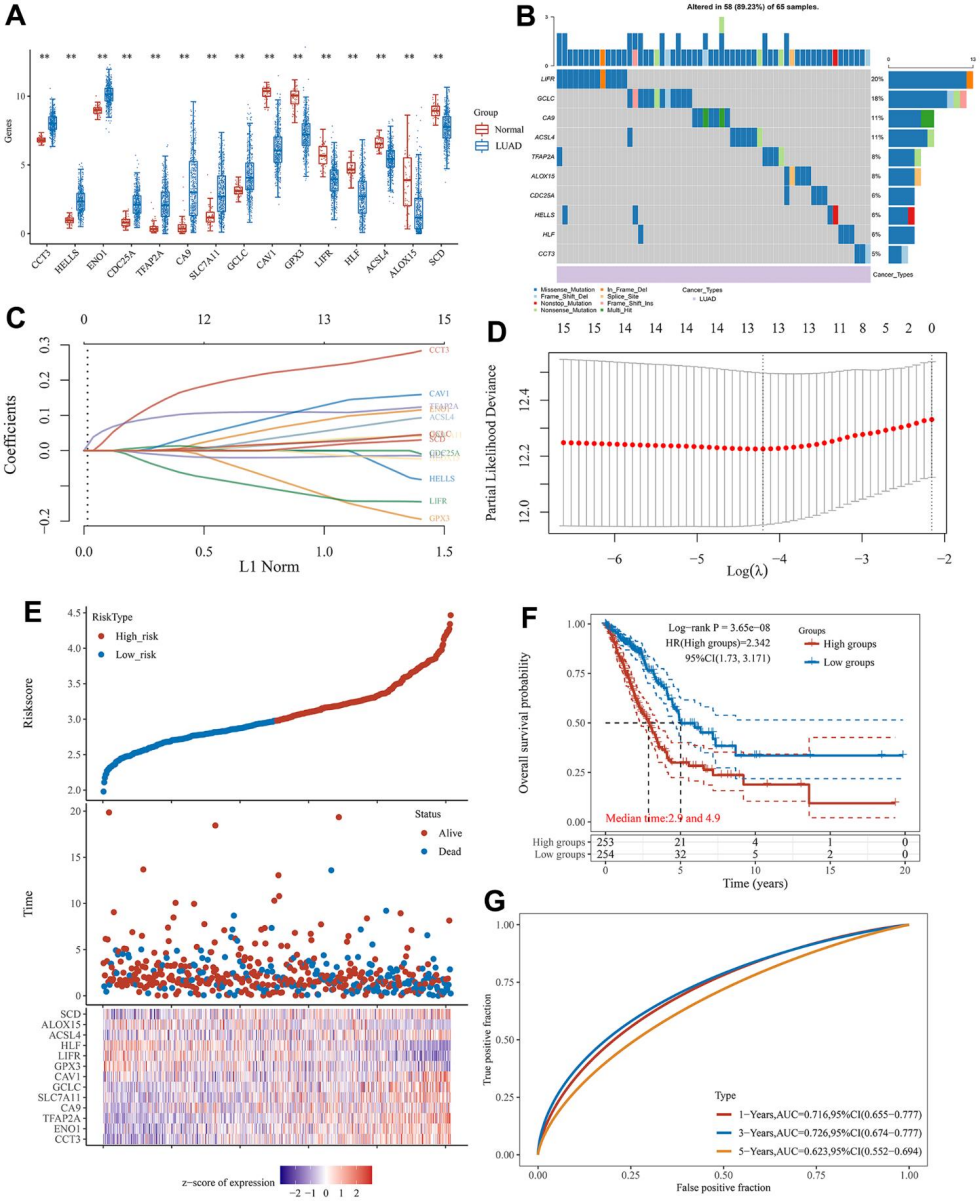
**The prognostic signature associated with CRFGs.** (**A**) Differential expression of 15 potentially prognostic CRFGs in LUAD samples and the normal samples. (**B**) Mutation landscape map of potentially prognostic CRFGs, showing SNV and genomic mutation types. (**C**, **D**) Prognostic signature established by LASSO Cox regression analysis. (**E**) Risk score distribution, patient survival status and CRFGs expression profile calculated by the prognostic signature. (**F**) Survival curves of LUAD patients in low- and high-risk groups. (**G**) ROC curves of the prognostic signature at 1, 3, and 5 years. *P < 0.05, **P < 0.01.

A univariate Cox regression analysis was performed on 44 CRFGs (Table 1), and 15 potentially independent prognostic genes were filtered out at a p-value <0.05, which showed differential expression in LUAD compared with normal tissues (Figure 3A). The mutational landscape of these 15 potentially prognostic genes was further analyzed using TCGA data to visualize the top 10 genes with the highest degree of mutation, with the results shown in Figure 3B, where the gene with the highest mutation density is the LIFR.

**Table 1:** OS-related univariate Cox regression analysis of 44 CRFGs.

**Uni_Cox**	**Pvalue**	**Hazard ratio (95% CI)**
TFAP2A	0.00005	1.23695(1.11607,1.37092)
CCT3	0.00025	1.51739(1.21406,1.8965)
CDC25A	0.00029	1.31458(1.13381,1.52417)
HLF	0.00069	0.8406(0.76035,0.92932)
ENO1	0.00154	1.44234(1.1498,1.80932)
LIFR	0.00168	0.80991(0.71011,0.92373)
GCLC	0.00247	1.14134(1.04769,1.24336)
SLC7A11	0.00891	1.11886(1.02855,1.2171)
HELLS	0.01939	1.20561(1.03069,1.4102)
CA9	0.02291	1.07088(1.00953,1.13597)
ACSL4	0.02331	1.19584(1.02461,1.39569)
CAV1	0.03002	1.11989(1.01101,1.24048)
SCD	0.04025	1.13687(1.00572,1.28513)
ALOX15	0.04032	0.90049(0.81464,0.99538)
GPX3	0.04371	0.87952(0.77637,0.99638)
TFRC	0.05972	1.12552(0.99517,1.27294)
GJA1	0.06981	1.1034(0.99206,1.22722)
TLR4	0.09284	0.87035(0.74022,1.02335)
MAP1LC3C	0.09328	0.90838(0.81195,1.01626)
NEDD4L	0.11818	0.87487(0.7398,1.0346)
CYBB	0.14379	0.92893(0.8415,1.02544)
ACSL1	0.16507	0.89012(0.75523,1.04911)
SLC39A8	0.17102	0.91088(0.79694,1.04112)
CDKN2A	0.19075	1.05277(0.97471,1.13708)
PTGS2	0.22003	1.04517(0.97392,1.12163)
CP	0.24934	1.04482(0.96971,1.12575)
ALOX5	0.28193	0.93514(0.8276,1.05664)
ETV4	0.40547	0.95551(0.85836,1.06366)
SLC40A1	0.43914	0.95399(0.84668,1.0749)
DDR2	0.4647	0.94663(0.81721,1.09656)
WWTR1	0.54997	1.05991(0.87583,1.28268)
HCAR1	0.58266	0.97332(0.88383,1.07188)
CYGB	0.60552	1.05051(0.87133,1.26654)
FBLN1	0.62394	0.96973(0.85759,1.09653)
JAM3	0.64954	0.964(0.823,1.12916)
LCN2	0.69188	1.01353(0.94835,1.0832)
ALAS2	0.71757	1.07262(0.73362,1.56827)
JUN	0.76688	0.97822(0.84569,1.13152)
PDK4	0.81162	0.98725(0.88837,1.09714)
IL6	0.82012	1.01138(0.91741,1.11497)
ZFP36	0.83636	1.01291(0.89686,1.14397)
LPCAT3	0.8883	0.98608(0.81083,1.19919)
ATF3	0.96585	0.9975(0.88946,1.11867)
EFEMP1	0.97338	0.99808(0.89172,1.11713)

### Establishment and validation of a prognostic signature associated with CRFGs

Based on these 15 potential independent prognostic genes of LUAD above, we performed LASSO Cox regression analysis to construct a prognostic signature associated with CFRGs. Finally, 13 genes were included in the prognostic signature (Figure 3C, 3D). The specific formulation of the prognostic signature: Riskscore=(0.2392)*CCT3+(0.0875)*ENO1+(0.1088)*TFAP2A+(0.0275)*CA9+(0.0302)*SLC7A11+(0.0251)*GCLC+(0.1293)*CAV1+(-0.1273)*GPX3+(-0.1325)*LIFR+(-0.017)*HLF+(0.0557)*ACSL4+(-0.0103)*ALOX15+(0.0144)*SCD. The risk plots and Kaplan-Meier survival analysis curves for the prognostic signature associated with CRFGs in Figure 3E–3G show that the high-risk group has a shorter survival time compared to the low-risk group. The 1-, 3-, and 5-year survival probability of the risk score was represented by the AUC values of 0.716, 0.726, and 0.623, respectively, in the TCGA cohort. These results indicate that the prognostic model constructed in this experiment possesses good stability.

**Figure 4:**
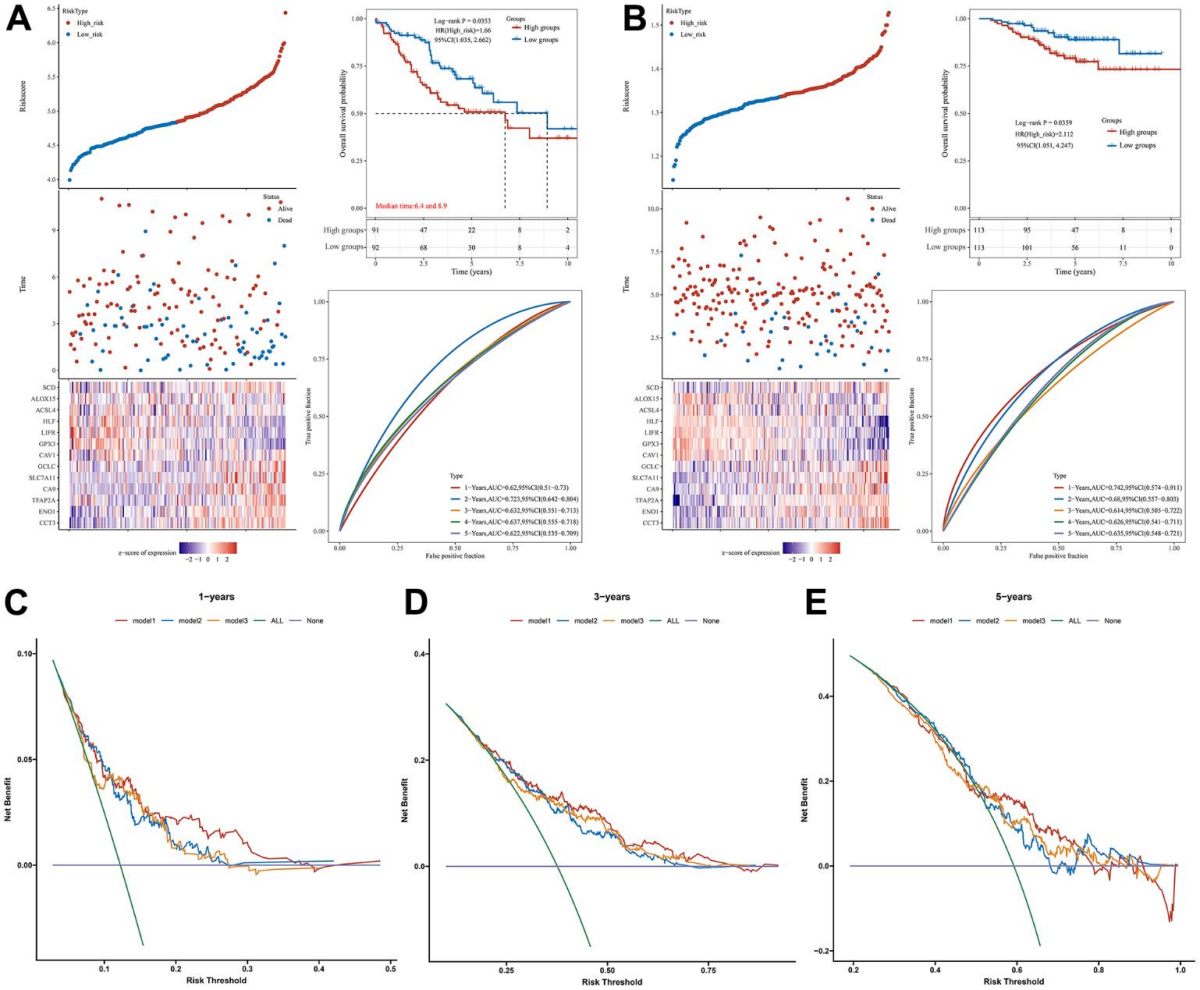
**Validation of the prognostic signature.** Validation of the prognostic signature in the external datasets (**A**) GSE41271 and (**B**) GSE31210. DCA curve was used to assess the clinical utility of the prognostic signature associated with CRFGs versus a single cuproptosis or ferroptosis model at (**C**) 1 year, (**D**) 3 years, and (**E**) 5 years of OS. Model1 represents the CRFGs-related model, Model2 represents the cuproptosis-related model, and Model3 represents the ferroptosis-related model.

To further validate the prognostic performance of our model, two datasets, GSE41271 and GSE31210, were selected in the GEO database as external validation cohorts for the prognostic performance of the model. As shown in Figure 4A, 4B, the validation results of both external cohorts demonstrate the generalizability and reliability of our constructed prognostic signature regarding CRFGs in LUAD. By DCA we found that the prognostic signature associated with CRFGs had better clinical utility compared with other single prognostic signatures for cuproptosis or ferroptosis (Figure 4C–4E).

**Figure 5:**
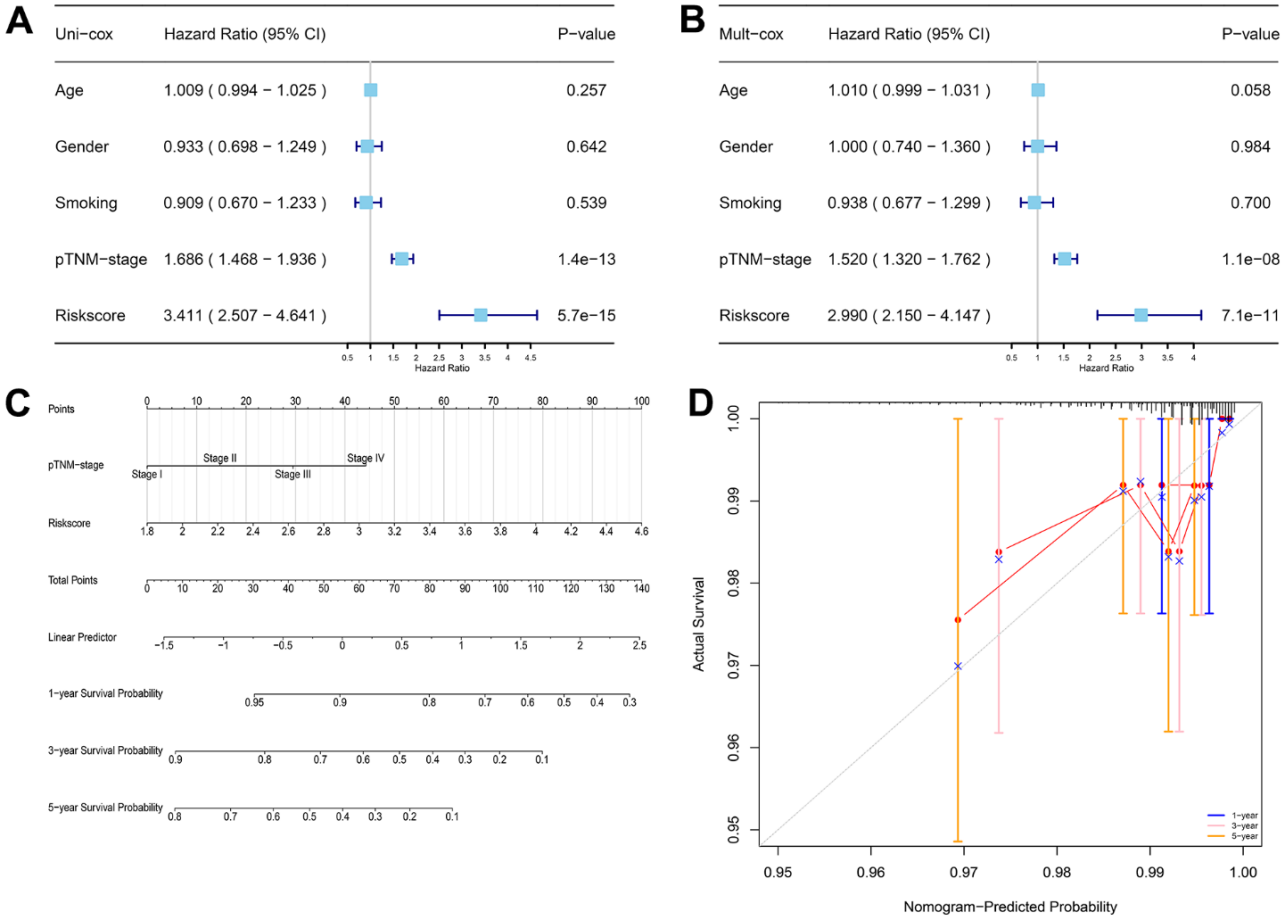
**The prognostic signature-related nomogram.** (**A**) Univariate Cox regression analysis. (**B**) Multivariate Cox regression analysis. (**C**) Nomogram for predicting the 1-, 3-, and 5-year OS of LUAD patients in the TCGA cohort. (**D**) Calibration curve to validate the established nomogram.

To further evaluate the potential clinical use of prognostic models associated with CRFGs, univariate Cox regression analysis (Figure 5A) and multivariate Cox regression analysis (Figure 5B) of prognostic signature together with associated clinical factors were performed and a nomogram was constructed (Figure 5C). The calibration curves of this nomogram show that the observed and predicted values have a high consistency (Figure 5D).

### Potential prognostic biomarkers

**Figure 6:**
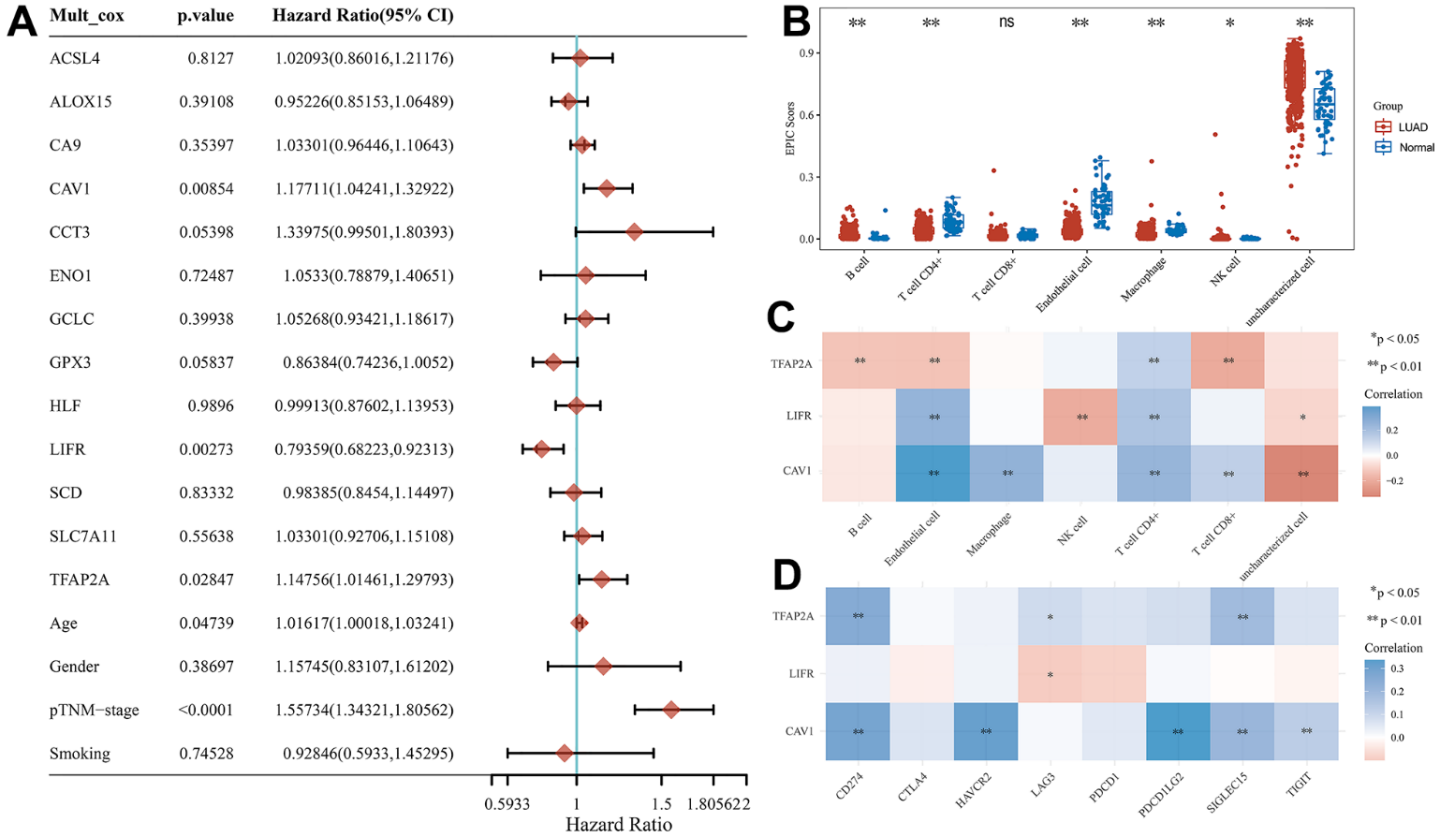
**Potential prognostic biomarkers acquisition and immunoassay for CRFG in LUAD.** (**A**) Multifactorial Cox regression analysis of CRFGs and associated clinical characteristics. (**B**) Immune scores in LUAD and normal tissues, the horizontal coordinate represents the types of immune cell infiltration and the vertical coordinate represents the distribution of immune scores in the two groups. (**C**) Heat map of correlation between potential prognostic biomarkers and immune scores, horizontal coordinates represent the type of immune cell infiltration, vertical coordinates represent genes. (**D**) Heat map of the correlation between the potential prognostic biomarkers and common immune checkpoints, horizontal coordinates represent immune checkpoints and vertical coordinates represent biomarkers.

To explore potential prognostic molecular mechanisms closely associated with LUAD progression, we performed a multivariate Cox regression analysis in the TCGA cohort combining age, gender, pTNM-stage, and smoking these clinical characteristics and 13 CRFGs. TFAP2A (p=0.02847), CAV1 (p=0.00854), LIFR (p=0.00273), age (p=0.04739), and pTNM-stage (p<0.0001) were shown to be potential prognostic factors closely associated with LUAD (Figure 6A). TFAP2A, CAV1, and LIFR were identified as potential prognostic biomarkers for further analysis.

### TME analysis

In the TCGA cohort, we calculated the degree of immune cell infiltration of seven common immune cells in LUAD tissues by the EPIC algorithm, and in Figure 6B, it can be found that the degree of infiltration of most immune cells was increased in LUAD compared to normal tissues, except for T cell CD8+. We then investigated the degree of immune infiltration of three potentially prognostic biomarkers and found that TFAP2A expression showed a negative correlation with the degree of B cell, Endothelial cell, and T cell CD8+ infiltration and a positive correlation with T cell CD4+ infiltration (Figure 6C).

We also researched the association between the eight universal immune checkpoints and the expression of these three potentially prognostic biomarkers, as shown in Figure 6D, where all three biomarkers have some association with the immune checkpoints.

### The core gene identification and analysis

**Figure 7:**
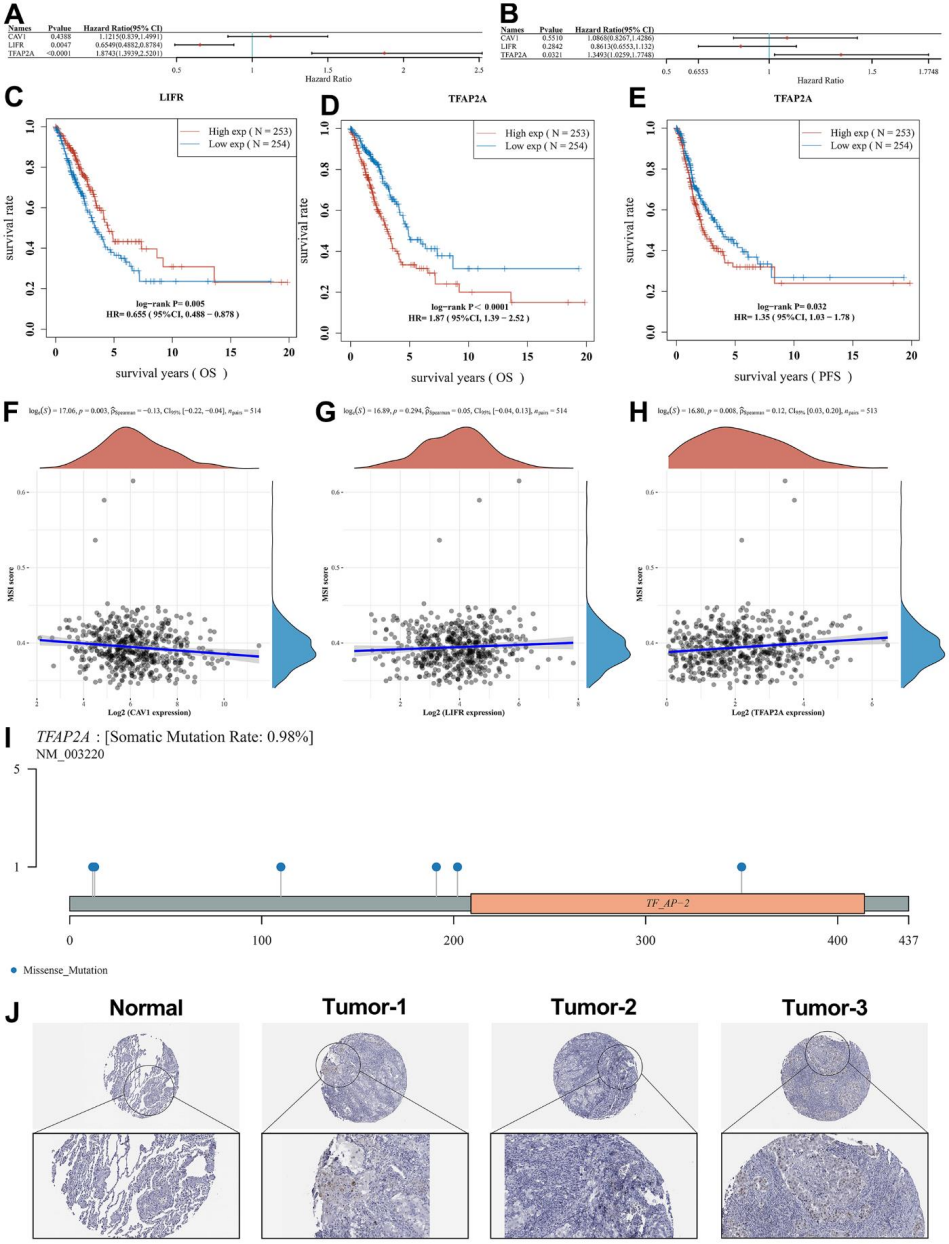
**Survival analysis of potential prognostic biomarkers and identification of the core genes.** Results of (**A**) OS and (**B**) PFS survival analysis for the potential prognostic biomarkers. (**C**) Relationship between LIFR expression and OS. (**D**) Relationship between TFAP2A expression and OS. (**E**) Relationship between TFAP2A expression and PFS. Correlation studies of potential prognostic genes with MSI, in which (**F**) CAV1 was significantly associated with MSI, while (**G**) LIFR was non-significantly associated with MSI as well as (**H**) TFAP2A was significantly associated with MSI. (**I**) Lollipop charts of the mutation landscape of TFAP2A, showing a somatic mutation rate is 0.98%. (**J**) Protein expression of TFAP2A in normal and LUAD tissues in pathological sections, indicating that TFAP2A was highly expressed in LUAD samples.

The results of the analysis of OS and PFS of the three biomarkers (Figure 7A, 7B) and Kaplan-Meier survival curves (Figure 7C–7E) showed that the high expression of TFAP2A was detrimental to survival in the LUAD patients.

High levels of Microsatellite instability (MSI), which predispose to the accumulation of mutations in cancer and an increase in tumor mutational burden (TMB), are detrimental to the control of cancer progression [25]. In this regard, we performed MSI scores for the three potential prognostic biomarkers. In Figure 7F–7H, it can be seen that CAV1 (p=0.003) and TFAP2A (p=0.008) expression showed a correlation with MSI score whereas LIFR (p=0.294) expression was not significantly correlated with it. Combining the results of the above analysis, we suggest that TFAP2A may act as a core gene and be closely related to the development of LUAD. Figure 7I shows the distribution of gene mutations in the TFAP2A. We also used pathological sections of TFAP2A protein expression to demonstrate core gene-related immunohistochemistry through the HPA database (Figure 7J), and it was seen that TFAP2A expression levels were significantly higher in LUAD tissues compared to normal tissues. These results further validate the upregulation of TFAP2A expression in LUAD tissues.

### Exploration and validation of lncRNA-related regulatory axis

**Figure 8:**
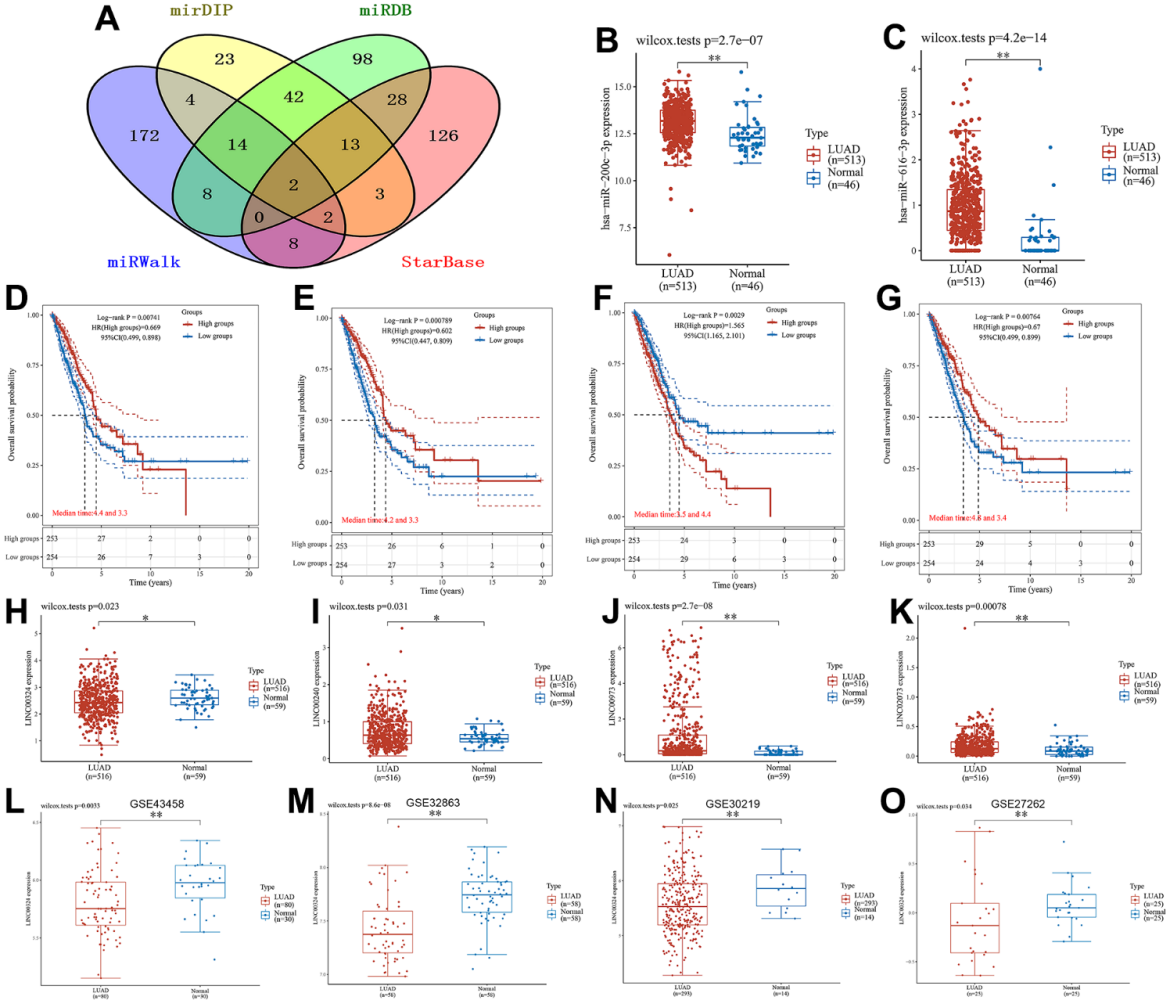
**Results of the lncRNA-related regulatory axis.** (**A**) Venn diagram using data from four databases: mirDIP, miRDB, miRWalk and StarBase. (**B**) Differential expression of miR-200c-3p, (**C**) miR-616-3p in LUAD samples and normal samples. Relationship between (**D**) LINC00324, (**E**) LINC00240, (**F**) LINC00973 and (**G**) LINC02073 expression levels and OS. Box plot of differential gene expression between (**H**) LINC00324, (**I**) LINC00240, (**J**) LINC00973, (**K**) LINC02073 in LUAD samples and normal samples. External dataset validation of expression differences of LINC00324. (**L**) GSE43458; (**M**) GSE32863; (**N**) GSE30219; (**O**) GSE27262.

To further elucidate the regulatory mechanisms associated with the core gene in LUAD, we conducted an exploration of the lncRNA-related regulatory axis. Relevant miRNAs about TFAP2A were obtained from the four databases (mirDIP, miRDB, miRWalk and StarBase) we selected for the search, and the specific results can be found in Supplementary Table 3. MiR-200c-3p and miR-616-3p were obtained from the comprehensive analysis as upstream miRNAs of TFAP2A (Figure 8A). In the TCGA cohort, both miRNAs showed increased expression compared to normal samples (Figure 8B, 8C). Next, the upstream lncRNAs of the obtained core miRNAs were predicted in the LncBase database, and then Kaplan-Meier analysis was performed to plot the survival curves. It should be noted that the prediction result of miR-616-3p was poor, so we selected miR-200c-3p as the subject of the follow-up study. As seen in Figure 8D–8G, the expression of four lncRNAs, LINC00324 (p=0.00741), LINC00240 (p=0.000789), LINC00973 (p=0.0029) and LINC02073 (p=0.00764), were closely associated with the prognostic outcome of LUAD. Their specific AUC values can be obtained from Supplementary Figure 1. We also analyzed the expression of the four lncRNAs mentioned above in LUAD and normal samples, and it could be found that they all had significant expression differences (Figure 8H–8K). After discussion and analysis of these four lncRNAs, we selected LINC00324, which has some research background and relevant expression results in the relevant GSE dataset, to establish the regulatory axis. To further validate the regulatory mechanism of the LINC00324/miR-200c-3p/TFAP2A regulatory axis constructed in this study, we selected four datasets (GSE43458, GSE32863, GSE30219, and GSE27262) in the GEO database as external validation cohorts. In Figure 8L–8O, it can be seen that our selected relevant lncRNA regulatory axis has good outcomes in the dataset. Based on the above validation results, we hypothesized that lncRNAs may play an important role in LUAD progression through the LINC00324/miR-200c-3p/TFAP2A regulatory axis.

## DISCUSSION

As one of the most frequently diagnosed cancers in the world, early diagnosis of lung cancer is a key research area to reduce its mortality [26]. The diagnostic tools currently used, such as Chest X-ray (CXR) and sputum cytology, are of interest in reducing the death rate of NSCLC [27]. However, the above methods have the shortcoming of low sensitivity for early screening of NSCLC [28, 29]. Therefore, there is an urgent need to find an early diagnosis method with high sensitivity and accuracy in the clinical treatment process. Compared to traditional screening methods, the addition of biomarker detection has provided new ideas for the early diagnosis of NCCLC in recent years [30, 31]. Numerous studies have shown that the establishment of cuproptosis-related gene signatures [32] and ferroptosis-related gene signatures [33, 34] have good prognostic value for LUAD. However, the correlation between CRFGs and LUAD remains to be investigated. In summary, we chose to build a CRFGs gene set to construct a prognostic signature and verify its ability to predict the prognosis of LUAD patients.

The GO and KEGG enrichment results of CRFGs showed that 44 CRFGs may be associated with biological processes such as the HIF1 signaling pathway, response to hypoxia, regulation of cellular response to stress, and defective intrinsic pathway for apoptosis. The above pathways have been found to be involved in the progression of LUAD. In LUAD cells, the glycogen branching enzyme (GBE1) is in the downstream of the HIF1 signaling pathway. It has been shown that the blockade of GBE1 signaling under hypoxic conditions can significantly inhibit the development of LUAD [35]. Dysregulation of the KEAP1/NRF2 stress response pathway promotes the development of LUAD [36]. Meanwhile, defects in apoptotic pathways are limiting for radiation therapy of NSCLC [37]. The above studies provide evidence from the side that CRFGs can act on LUAD with important potential molecular mechanisms that can support our in-depth study.

Further univariate Cox regression and LASSO Cox regression analyses optimized to establish the prognostic signature associated with CRFGs showed good performance in predicting the prognosis of LUAD patients. As seen in the SNV analysis figure, genes such as LIFR, GCLC, CA9, ACSL4, and TFAP2A have high mutation rates in LUAD, which play an important role in the overall survival of such patients. Previous studies have proven that the expression level of LIFR correlates with disease progression and survival of patients [38]. GCLC, a new LUAD prognostic biomarker, whose expression is upregulated accelerates the process of ferroptosis and inhibits the proliferation and invasion of LUAD cells [39]. Meanwhile, TFAP2A can regulate the miR-16 family/TFAP2A/PSG9/TGF-β signaling pathway [40] by inducing the expression of KRT16 [41], ITPKA [42], and other oncogenes to affect the development, migration and invasion of LUAD. In addition, it has been suggested that TFAP2A can regulate the ferroptosis of gallbladder cancer cells through the Nrf2 signaling axis, so TFAP2A may act as a regulatory factor of ferroptosis [43]. The prognostic signature established with TCGA-LUAD data, validated by external datasets in the GEO database, and the comparison of nomogram and DCA can indicate that our prognostic signature related to CRFGs has better results and greater advantages in predicting the prognosis of LUAD patients, which has some significance for clinical application.

The TME occupies an important position in tumor-related research, and the evaluation of the immune system facilitates the development of personalized immunotherapy strategies for LUAD patients [44]. It has been suggested that the ferroptosis score and cuproptosis-related gene score are important for TME cell infiltration in LUAD patients and assist in assessing the effects of immunotherapy [18, 45]. It was suggested that the degree of intra-tumor infiltration of immune cells and the immune checkpoint correlation could be used for the development of prognostic markers for LUAD [46, 47]. We performed a multivariate Cox regression analysis and found that TFAP2A, CAV1, and LIFR were of greater research potential among the above prognostic biomarkers. Immune infiltration analysis showed that TFAP2A, CAV1, and LIFR were highly correlated with most immune cells in LUAD. Similar findings also pointed out that TFAP2A may act as a transcription factor for SLC7A5 and affect the expression of SLC7A5 in LUAD cells, thus causing alterations in immune-related gene expression and immune cell infiltration [48]. The LIF/LIFR axis could be a valuable clinical target for the regulation of multiple immune cells in TME [49], for example, by affecting the proliferation of CD4(+) and CD25(+) regulatory T cells [50]. The deletion of CAV1, a membrane-intrinsic protein with inhibitory effects on LUAD, in TME may affect the survival of cancer cells [51]. Current studies have shown that CAV1 can act as an immune-related biomarker for LUAD, with positive correlations between different types of immune infiltration [52]. In addition, immune checkpoint blockade (ICB) therapy has been widely used in the treatment of NSCLC [53, 54], and there is an urgent need to find effective biomarkers to identify patients with LUAD who are sensitive to immune checkpoint inhibitors. Our study found that TFAP2A and CAV1 have better responsiveness to common immune checkpoints, providing insight into ICB therapy for LUAD patients.

There are extensive and complex inter-regulatory interactions between coding and non-coding RNAs [55]. In recent years, an emerging RNA crosstalk-competing endogenous RNA (ceRNA) hypothesis has been proposed [56] and an increasing number of studies have supported this kind of it. It has been shown that ceRNA can play a regulatory role as a natural endogenous miRNA sponge competing for binding miRNA [57]. As a type of ceRNA, lncRNA has received extensive attention on the mechanism of reverse regulation by competing with mRNA to bind miRNA [58]. In parallel, it was found that lncRNA can participate in the multilayered regulatory circuitry of cancer cell biological behavior through the ceRNA network, showing potential therapeutic and prognostic value [59, 60]. Accordingly, we further performed OS and PFS analyses on TFAP2A, CAV1, and LIFR, which are the key genes in CRFGs, and constructed a CRFGs-related ceRNA network by comparing and selecting TFAP2A with the best prognostic performance. Through various database screening and lncRNA prognostic analysis, we finally found that CRFGs may affect LUAD progression through LINC00324/miR-200c-3p/TFAP2A regulatory axis. In the available studies, LINC00324 has been shown to play a role through the regulatory axis in a variety of cancer cells [61] and is considered a promising marker for tumor prognostic properties. Multiple bioinformatic analyses identified it as a ferroptosis and iron-metabolism related lncRNA signature for LUAD, involved in the construction of the prognostic signature [62] and coregulatory axis [63]. We demonstrated through substantial data in the TCGA-LUAD and GEO databases that LINC00324 acts as a protective factor in the development of LUAD and that its high expression favors the prognosis of LUAD patients, consistent with the findings of related studies [64]. Meanwhile, we also found that in this lncRNA regulatory axis, both miR-200c-3p and TFAP2A expression levels were increased in LUAD patients, while LINC00324 expression levels were decreased. Combined with the relevant prognostic survival curve results, we propose the hypothesis that LINC00324 can be regulated by suppressing downstream gene expression. We believe that the decreased expression of LINC00324 in LUAD increased the level of miR-200c-3p, which promoted the expression of TFAP2A, while LINC00324 may also regulate the expression of downstream TFAP2A through a mechanism of competitive binding with miR-200c-3p, thus affecting the biological behavior of tumor cells and providing new approaches and ideas for clinical targeting therapy and targeted drug development of LUAD.

Unfortunately, there are some limitations in our current study. The research was carried out based on the existing bioinformatics database, and the reliability of the information in the database influenced our results. Furthermore, although we used a large amount of external data from the GEO database to demonstrate the stability and reliability of our results, there was still a lack of *in vivo* or *in vitro* experimental validation. In the future, we will further explore the efficacy and specific mechanisms of the CRFGs-related LINC00324/miR-200c-3p/TFAP2A regulatory axis acting on LUAD cells in depth through relevant experiments.

## CONCLUSIONS

In conclusion, we constructed a CRFGs-related LUAD prognostic signature in this study, which exerted a good prognostic ability to predict LUAD patients. In particular, we identified three more prognostic biomarkers (TFAP2A, CAV1, and LIFR) with more investigational value to provide new ideas for immunotherapy in LUAD patients. By exploring the lncRNA regulatory axis, we also found that the CRFGs-related LINC00324/miR-200c-3p/TFAP2A axis may be involved in the progression of LUAD, and the specific efficacy of its role remains to be verified by further experiments.

## MATERIALS AND METHODS

### Public data acquisition and pre-processing

For Ferroptosis, the GeneCards database (https://www.genecards.org) was searched for related genes using the keyword “Ferroptosis” and the filtering condition was set to a “Relevance score” greater than or equal to 1. Cuproptosis-related genes were obtained from a previous study in a summary collection [13]. RNA-sequencing expression (level 3) and corresponding clinical information of LUAD were obtained from The Cancer Genome Atlas (TCGA) database (https://portal.gdc.cancer.gov/). A total of 516 LUAD patients and 59 paraneoplastic samples from the TCGA database were involved in this study. The microarray data sets (GSE41271, GSE31210, GSE32863, GSE43458, GSE30219, and GSE27262) were extracted from Gene Expression Omnibus (GEO) database (https://www.ncbi.nlm.nih.gov/geo/), downloaded in MINiML format, and used the removeBatchEffect function of the limma package in the R software to remove batch effects.

### Acquisition of CRFGs in LUAD and related gene enrichment analysis

The genes related to ferroptosis and cuproptosis were imported into the STRING database (https://string-db.org) to construct protein-protein interaction (PPI) networks, from which CRFGs were obtained, and then the PPI networks were visualized in Cytoscape_v3.8.2. The “Limma” package in R software was used to explore differentially expressed genes (DEGs) in LUAD tissues versus normal tissues. Fold change and corrected p-values were used to plot the Volcano plot heatmap. Using the “pheatmap” package to draw heat maps. Subsequently, the common genes of CRFGs and LUAD-DEGs were acquired using Draw Venn Diagram (http://bioinformatics.psb.ugent.be/webtools/Venn/) for further analysis. To explore the potential biological functions and pathways of action of CRFGs, GO and KEGG enrichment analysis of common genes was performed using Metascape (https://metascape.org), setting p-values <0.05.

### Obtaining and analyzing potential prognostic genes in CRFGs

The common genes were subjected to univariate Cox regression analysis, and the P values, hazard ratio (HR), and 95% confidence interval (CI) values of each gene were displayed by the “forestplot” package in R software, and statistically significant (p-values <0.05) genes were selected as potential independent prognostic factors for further analysis. The gene expression differences of independent prognostic genes in LUAD tissues and normal tissues were then statistically analyzed using the “ggplot2” package in R software and tested by the Wilcox-tests. Mutation information of these potential prognostic genes in LUAD was obtained through the GSCALite online website (http://bioinfo.life.hust.edu.cn/web/GSCALite/), and the top ten genes with the greatest degree of mutation were visualized.

### Construction and validation of a prognostic signature related to CRFGs

The above potential independent prognostic genes were used to construct a CRFGs prognostic signature by LASSO Cox regression analysis using the “survival” and “glmnet” packages in R software. In the prognostic signature, a risk score is used for presentation. The risk score formula is shown here: risk score = (coefficient mRNA1 * expression of mRNA1) + (coefficient mRNA2 * expression of mRNA2) +… + (coefficient mRNAn * expression mRNAn). Then, all samples were divided into high-risk and low-risk groups using the median of the risk scores and the corresponding survival curves were plotted using Kaplan-Meier analysis and tested by the Log-rank method. To predict the accuracy of prognostic signature for LUAD patients, the time-related ROC curve should be completed. Meanwhile, two expression microarray datasets, GSE41271 and GSE31210, selected from the GEO database, were used for external validation of the prognostic signature to discern the accuracy of the risk score.

Finally, to further confirm the stability of our risk score models, we constructed DCA using the “ggDCA” package in R software. The model established in this study was compared with the previous LUAD-related models for ferroptosis [33] and cuproptosis [65].

### Establishment of nomogram

To evaluate the prognostic value of the model, the prognostic signature developed in this experiment and the clinical factors related to LUAD such as age, gender, pTNM-stage, and smoking, were subjected to univariate Cox regression and multivariate Cox regression analyses, and forest plots were constructed using the “forestplot” package in R software. Finally, based on the results of the multivariate Cox proportional risk analysis, the nomogram was constructed using the “rms” package to predict the overall survival (OS) of LUAD patients at 1, 3, and 5 years. The “rmda” R package was then used to build calibration curves and to evaluate the degree of consistency between the OS predicted rate and the actual OS rate by our established nomogram.

### Correlation immunoassay of potential prognostic biomarkers

The EPIC algorithm in the R “immunedeconv” package was used to score the degree of immune cell infiltration in LUAD, and the results were visualized using the “ggplot2” and “pheatmap” packages in the R software. A multivariate Cox regression analysis was performed on the above prognostic risk score models for genes and relevant clinical factors, using the “forestplot” package to construct a forest plot to identify potential prognostic biomarkers, and then the R package “pheatmap” was used to show the correlation of potential prognostic biomarkers with immune scores and their expression correlation with common immune checkpoints.

### Survival analysis of related genes, MSI and immunohistochemistry

OS and progression-free survival (PFS) curves of potential prognostic biomarkers in high and low expression groups were plotted using the Kaplan-Meier approach and tested by Log-rank. The correlation of potential prognostic biomarker expression with MSI scores was assessed by the “ggstatsplot” package. After combining univariate Cox regression analysis and multivariate Cox regression analysis including relevant clinical factors, we also combined OS and PFS curves with MSI scores as a way to further screen the core gene closely associated with LUAD progression. The “maftools” package was used to visualize the distribution of mutations in core genes in LUAD. Also, we used the Human Protein Atlas (http://www.proteinatlas.org) to show the pathological profiles of the immunohistochemistry of the core gene.

### lncRNA-related regulatory axis acquisition and external validation

The upstream miRNA information of the core gene was collected from four databases, mirDIP (ophid.utoronto.ca/mirDIP), miRDB (https://www.mirdb.org), miRWalk (mirwalk.umm.uni-heidelberg.de) and StarBase (https://ngdc.cncb.ac.cn/databasecommons/database/id/169), after which the critical miRNA of the gene was obtained using Venny 2.1.0 (bioinfogp.cnb.csic.es/tools/venny). The “ggplot2” package was used to statistically analyze the differences in the expression of these core miRNAs in LUAD tissues and normal tissues and tested by the Wilcox-tests. After that, the upstream lncRNAs of the core miRNA were predicted using the LncBase database (https://diana.e-ce.uth.gr/lncbasev3). The Kaplan-Meier analysis method was used to plot survival curves regarding the high and low expression of lncRNAs, which were then tested by Log-rank with a screening condition of p-value <0.01. The data of LUAD were obtained from the TCGA database, and then the “ggplot2” package was used to statistically analyze the screened upstream lncRNAs and compare their expression differences in LUAD tissues and normal tissues. On balance, we chose lncRNAs with a certain research base and analytical results in the database to construct the regulatory axis, and to serve as the target for subsequent analysis. Finally, for the mechanism of action of the selected lncRNA-related regulatory axis we chose the external validation sets (GSE43458, GSE32863, GSE30219 and GSE27262) for validation and tested them using the Wilcox-tests.

### Statistical analysis

The above statistical analysis was performed using R software (version 4.0.3) and the associated database. The p-value < 0.05 was considered statistically significant in this study.

### Data availability statements

The original contributions presented in the study are included in the article or Supplementary Material. Further inquiries can be directed to the corresponding authors.

## Supplementary Material

Supplementary Figure 1

Supplementary Table 1

Supplementary Table 2

Supplementary Table 3
