# Supplementary material for: Identification and integration analysis of a novel prognostic signature associated with cuproptosis-related ferroptosis genes and relevant lncRNA regulatory axis in lung adenocarcinoma
Source: Aging (Albany NY). 2023 Mar 3;15(5):1543–63. doi: 10.18632/aging.204561 (PMC10042693; doi:10.18632/aging.204561)
Supplement: Supplementary Figure 1 [file aging-15-204561-s001.pdf]

SUPPLEMENTARY FIGURE

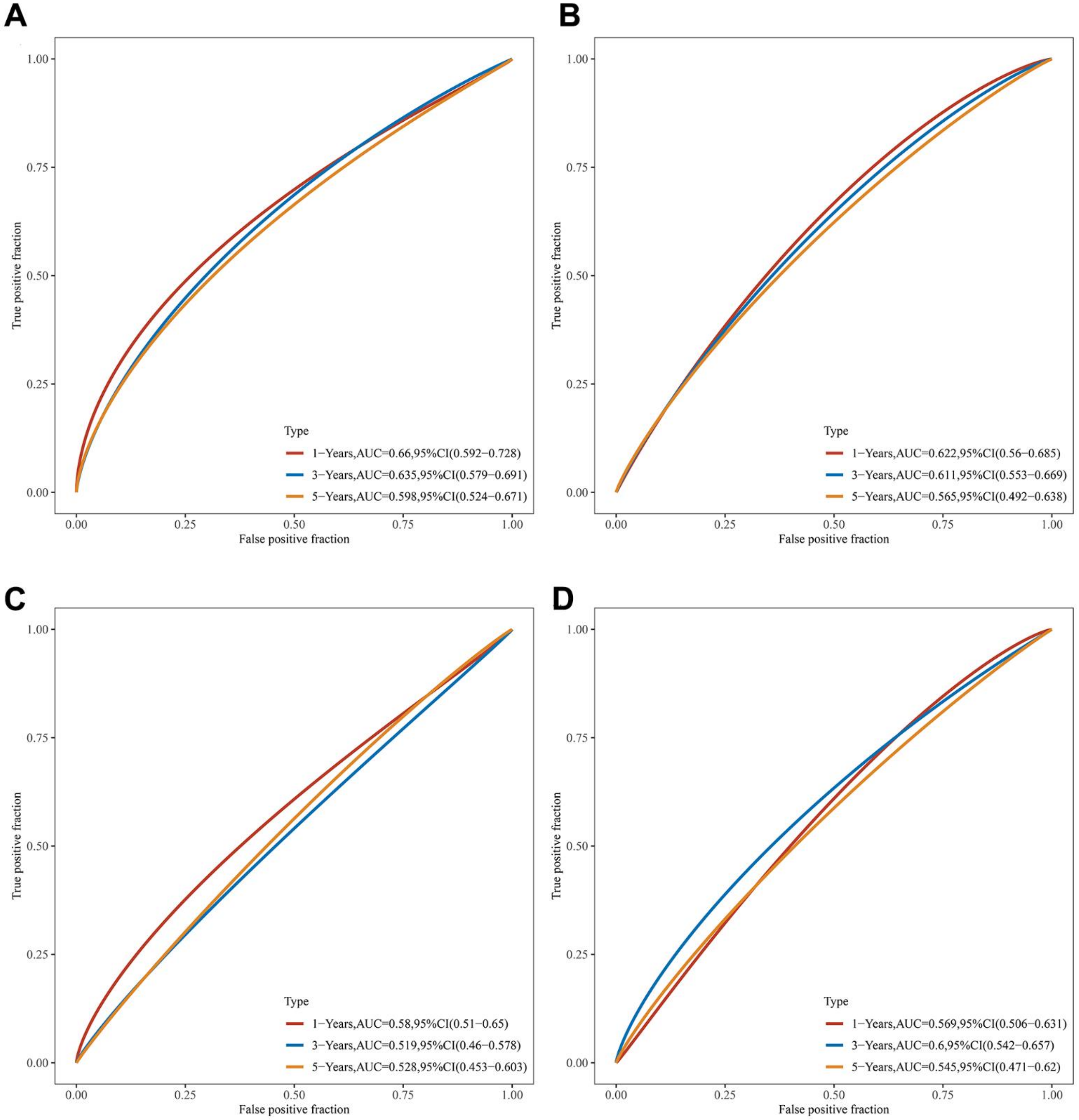

Supplementary Figure 1. ROC curves on LUAD prognosis for (**A**) LINC00324, (**B**) LINC00240, (**C**) LINC00973, and (**D**) LINC02073.
